# Psychological distress in a sample of Moroccan prisoners with drug-dependence

**DOI:** 10.1177/0306624X211010286

**Published:** 2021-04-21

**Authors:** Anis Sfendla, Björn Martinsson, Ylva Filipovic, Meftaha Senhaji, Nóra Kerekes

**Affiliations:** 1 Higher Institute of Nursing Professions and Health Techniques, Errachidia, Morocco; 2 Department of Biology, Faculty of Sciences, Abdelmalek Essaâdi University, Tetouan, Morocco; 3 Department of Biology, Faculty of Sciences and Techniques, Moulay Ismail University, Errachidia, Morocco; 4 Department of Health Sciences, University West, Trollhättan, Sweden

**Keywords:** brief symptom inventory, drug use disorders identification test, mental illness, Morocco, prison inmates, substance use

## Abstract

Research regarding mental illness and drug addiction among inmates in Morocco requires increased knowledge; previous literature reported that prisoners suffer from severe psychological distress. The present study aimed to provide information about Moroccan prisoners’ psychological distress and define the differences in psychological distress levels among inmates with and without drug-dependence. A sample of 177 male inmates completed a set of surveys, including the Drug Use Disorders Identification Test (DUDIT) and the Brief Symptom Inventory (BSI). The “Drug dependence” group scored significantly higher psychological distress levels in each of the BSI domains. The strongest differences were measured in the General Severity Index (GSI), hostility, and depression scales. Moroccan prison inmates have high psychological distress, and those with drug-dependence have even higher. There is a need of psychiatric assessment, selection, and care possibilities in prison inmate populations.

## Introduction

Global reports indicate an increase in mental illness and drug use in Arabic speaking countries, despite the general culture’s disapproval of the use of drugs (Arfken & Ahmed, 2016). This increase can be explained by a rapidly evolving society and overall modernization. There is an urgent need for published research regarding mental health and substance use in developing countries (Gaferi et al., 2013).

Epidemiological studies on psychiatric disorders are relatively rare in developing countries, especially in Arab ones (Saxena et al., 2006). According to the few completed studies, a substantial number of people in these countries suffer from psychiatric problems. The latest “Global Burden of Disease and Risk Factors” report (Lopez et al., 2002) revealed that neuropsychiatric disorders account for 9.8% of the total burden of diseases in low- and middle-income countries. There is no insight on the burden caused by mental disorders in the Arab world specifically. In Morocco, for instance, only a few studies have been focused on mental health. There are two describing the prevalence of mental disorders in the general population (Chabaud et al., 2017; Kadri et al., 2010), those exploring mental health profiles among high school students (Zarrouq et al., 2016; Zouini et al., 2019), and another two among incarcerated and delinquent population (Lahlou et al., 2010; Sfendla et al., 2018b).

Mental ill-health and psychiatric morbidity have been observed to be higher among prisoners compared to the general population (Andersen, 2004; Joukamaa, 1995). While some research has found that fewer of the inmates report a psychiatric diagnosis when entering the justice system/prison and that mental illness develops during incarceration (Slade et al., 2016), recent reviews in the subject prove that mental disorders are overrepresented and substance abuse is increased many fold in prisoners (Fazel et al., 2016). The most frequently reported psychiatric diagnoses among prisoners are personality disorders, mood disorders, addiction, and neurodevelopmental disorders (Fazel & Danesh, 2002; Fazel et al., 2016; Sansone & Sansone, 2009).

A meta-analysis revealed that drug abuse and dependence prevalence estimates ranged between 10% to 48% in men and 30% to 60% in women upon their arrival to prison (Fazel et al., 2006). Both outside and inside prisons, substance use/dependency and affective disorders have been rated with high comorbidity (Andersen et al., 1996; Bland et al., 1990). Furthermore, Fazel and Danesh, in their systematic review including 22,790 prisoners from 12 countries, revealed that prisoners were several times more likely to have depression and psychosis, while antisocial personality was ten times more often reported compared to the general population; 3.7% of the prisoners had psychosis, 10% had major depression, and 65% were diagnosed with personality disorder (Fazel & Danesh, 2002).

The mental health situation and prevalence of psychiatric conditions in developing countries among prisoners remains unknown. The limited research in the field can explain the lack of awareness among policymakers about mental health disorders. The previous systematic review reported only three studies from non-western countries (Fazel & Danesh, 2002). The mental health conditions of inmates remain a major research challenge; the high prevalence of mental disorders among this specific group has been confirmed by previous research (Iversen et al., 2014; Osasona & Koleoso, 2015). It is important to emphasize that these findings of psychiatric disorders and substance use in prison populations originated from Western cultures and developed countries. At present, only a limited number of studies have described the mental health of prison inmates in developing countries (Gharaibeh & El-Khoury, 2009). One of the first studies, including Moroccan prison inmates—as part of a high-risk population for drug dependence—revealed that about 40% of their study population had diagnosed psychiatric conditions (Sfendla et al., 2018b).

In Morocco, the total prison population increased from 61,405, or 189 prisoners per 100,000 inhabitants, in 2011 (Walmsley, 2011) to 76,000, or 222 prisoners per 100,000 inhabitants, in 2015, thereby ranking Morocco in the third place among Arab countries and the sixth in Africa (Walmsley, 2015a). Morocco ranks among the top 21 countries worldwide in terms of the size of its prison population and the first compared to North African countries (Walmsley, 2015b; World Prison Brief, 2019). The prisoners’ psychiatric and social follow-up is insufficient and suffers from several constraints (World Health Organization, 2006). These shortcoming manifest first and foremost by a minimal number of nursing staff, composed of a single psychiatrist per institution, and above all by the absence of a permanent multidisciplinary structure comprising physicians and psychiatric, psychotherapy and social assistance (Gharaibeh & El-Khoury, 2009; Okasha et al., 2012).

Substance use can be linked to various problems, including psychological distress and health problems, which in turn can cause significant costs and harm (Esmaili, 2018; Khalooei et al., 2016). The relationship between substance use and mental ill-health is bi-directional. On the one hand, mental illnesses such as anxiety, depression, psychosis, and schizophrenia are often linked to substance use (Bovasso, 2001; Fazel et al., 2009; Roberts et al., 2007; Ross et al., 1988; Sfendla et al., 2018b). In this case, substance use/abuse is initiated to aim a self-medication process, where people tend to use drugs to ease distress (Khantzian, 1985; Sinha, 2008). On the other hand, the use of various substances (e.g., cocaine, opioids, and cannabis) can cause psychiatric disorders; for instance, opioid use can lead to anxiety disorder (Arunogiri & Lubman, 2015; McHugh, 2015) and marijuana dependence can lead to attention deficit hyperactivity disorder (ADHD) (Wilens et al., 2011).

This study aims to (1) provide information about the mental health/ill-health of a Moroccan prison population with the help of a self-reported measure of psychological distress, and to (2) define the differences in the levels of psychological distress in inmates with “Drug dependence” and those with “No drug dependence”.

## Participants and Methods

### Study Design

The data were collected from male prison inmates in Meknes, Morocco. A cross-sectional design was implemented in the present study. The study is part of the “Mental and Somatic Health without borders-project (MeSHe)” with the general aim to identify culture-specific patterns in predictors of persistent mental ill-health, using the MeSHe survey (www.meshe.se). The surveyed inmates were recruited from the “Toulal 2” prison in Meknes, Morocco. The study included 177 male participants reflecting 7.2% of the total number of prisoners (2,453) incarcerated at the time of data collection. The MeSHe survey included beside other validated instruments the Brief Symptom Inventory (BSI) and the Drug Use Disorders Identification Test (DUDIT). The ability to read and write in Arabic was required for participation in the study. The survey was carried out with 20 to 30 inmates in classrooms provided by the prison administration. A member of the research team was present while the participants answered the questionnaire, to provide any clarification if required. The average time needed to complete all components of the MeSHe survey was about 60 minutes.

### Study Population

The sample included (*n* = 177) male inmates. Three participants did not report answer on education, job, and marital status. The mean age was 30.88 years old (SD = 10.66), age range was (Min = 15, Max = 92) with a median of 28. The reported education status was: 19.2% (*n* = 34) for higher education (college and university), 32.2% (*n* = 57) for high school, 29.9% (*n* = 53) for secondary school and 15.8% (*n* = 28) for elementary school, while 1.1% (*n* = 2) had no educational qualifications. Over three-fourths of the participants, 76.8% (*n* = 136), had been employed at the time of their incarceration. With regard to marital status, 57.1% (*n* = 101) were single, 29.4% (*n* = 52) were married, 5.6% (*n* = 10) were divorced, 3.4% (*n* = 6) had a partner, 2.3% (*n* = 4) were remarried, and 0.6% (*n* = 1) reported being separated.

### Instruments

#### Drug Use Disorders Identification Test

The Drug Use Disorders Identification Test (DUDIT) (Berman et al., 2005) was developed to identify people with drug-related problems. Its purpose is to recognize patterns regarding drug use and associated problems in daily life. The instrument’s psychometric characteristics have been tested in clinical and general populations in several countries (Hildebrand, 2015). The instrument consists of eleven items that identify characteristics related to drug use. Items one to nine are rated on a five-point Likert-scale (0-4), while items ten and eleven are rated on a three-point scale (0-2-4). Consequently, the total score can range from 0 to 44. In the present study, the Arabic version of the DUDIT was used. The validity and reliability of DUDIT were previously supported (Berman et al., 2005). The present study also supports the instrument’s reliability among inmates (Sfendla et al., 2017). For the Arabic version of the DUDIT, a specific cutoff (≥3 points) has been determined to identify individuals with drug dependence (Sfendla et al., 2017).

#### Brief Symptom Inventory

The Brief Symptom Inventory (BSI) is a self-reported measure of psychological distress (Derogatis & Melisaratos, 1983). The instrument consists of 53 questions clustered into nine primary symptom domains: anxiety, somatization, psychoticism, paranoid ideation, obsessive-compulsiveness, hostility, phobic anxiety, depression, and interpersonal sensitivity. The items assess the prevalence of specific symptoms and are rated on a five-point Likert scale ranging from 0 (not at all) to 4 (extremely). The BSI has previously shown good psychometric features (Franke & Derogatis, 2000), with high internal consistency among general (Zouini et al., 2019) and prison population (Boulet & Boss, 1991; Derogatis & Savitz, 1999; Dirkzwager & Nieuwbeerta, 2019), and also high test-retest reliability (Derogatis & Spencer, 1982). In the present study the nine primary domains and the Global Severity Index (GSI) were analyzed. The GSI is based on the sum of ratings on all 53 items. It measures the overall psychological distress level (Derogatis & Melisaratos, 1983). Five domains and the GSI had acceptable internal reliability with Cronbach alpha between 0.7 (hostility domain) and 0.96 (GSI), while the domains of psychoticism (α = 0.65) and phobic anxiety (α = 0.64) had close to acceptable reliability.

### Statistical Analysis

The study sample and the BSI primary domains were analyzed with descriptive statistics, including means (M), standard deviations (SD), range (Max-Min), and frequencies.

The previously validated cut off (≥3 points) for the Arabic DUDIT was used to place the participants in either of two groups: inmates scoring three or more points in the DUDIT were placed in the “Drug dependence” group, while those scoring less than three points were placed in the “No drug dependence” group. Each question in the BSI questionnaire was coded to its related domain and divided by the total number of questions in each domain (questions divided by the number of items).

Violation of the normality distribution assumption for the whole sample was supported by the Shapiro–Wilk test reaching a significant value (*p* < .05). Therefore, the non-parametric Mann–Whitney *U* test was used to analyze the difference between the “Drug dependence” and the “No drug dependence” groups.

Cohen’s *r* effect size for each BSI domain was calculated by dividing the *z*-values with the square root of the number of cases (*n*). The Mann–Whitney *U* test obtained the *z*-values. Cohen’s guidelines for *r* were followed during its interpretation, where a large effect size is defined by *r* ≥ 0.5, a medium effect size by *r* ≥ 0.3, and a small effect size by *r* ≥ 0.1 (Fritz et al., 2012). The Cronbach Alpha (α) reliability test was performed for each BSI domain to measure the questionnaire’s internal consistency. All analyses were performed using SPSS for Windows (IBM) version 24.0.

### Ethics Approval and Consent to Participate

The data collection was approved by the “Directorate-General of Prison Administration and Rehabilitation” (case identifier number: 13993), Rabat, Morocco. Informed consent was obtained from all individual participants included in the study. Participation was voluntary and anonymous. The study was carried out in accordance with the 1964 Helsinki declaration and its later amendments (World Medical Association, 2013). The objectives of the “Mental and Somatic Health without borders” survey were explained both in writing and orally. The inmates were assured that their answers would not and could not affect their current sentence. Those not wishing to complete the full survey could interrupt their participation and leave the room freely without giving a reason.

## Results

### Mental Health Profile of Moroccan Prisoners

The surveyed Moroccan male inmates’ overall psychological distress level reached a GSI of 1.19 (*SD* = 0.69). The most frequently reported symptoms were those captured by the paranoid ideation domain, such as symptoms of suspiciousness and distrust toward others and fear regarding the intentions of others, and by the obsessive-compulsiveness domain, such as symptoms of trouble remembering things or concentration, difficulty making decision and persistent thoughts of checking one’s actions. The least frequently reported symptoms were feeling fear of crowds, open spaces and specific situations (phobic anxiety domain), signs of irritability, impulsivity, and aggression (hostility domain) and physical complaints originating from psychological distress (somatization domain). The detailed scores of the nine primary symptom domains and the GSI scale are summarized in Table 1.

**Table 1.** Moroccan prisoners’ mental health profile operationalized as psychological distress and measured by the Brief Symptom Inventory (BSI), represented by means (*M*), and standard deviation (*SD*) in each subscale.

BSI domains	*M*	*SD*	Range (max–min)
Anxiety	1.21	0.82	0.0–4.0
Somatization	1.08	0.75	0.0–3.4
Psychoticism	1.25	0.80	0.0–3.8
Paranoid ideation	1.52	0.92	0.0–4.0
Obsessive-compulsiveness	1.50	0.81	0.0–3.8
Hostility	1.07	0.78	0.0–3.4
Phobic anxiety	0.82	0.71	0.0–3.4
Depression	1.22	0.90	0.0–3.8
Interpersonal sensitivity	1.28	1.00	0.0–4.0
GSI	1.19	0.69	0.0–3.4

### Drug Dependence and Mental Health

Table 2 compares the nine primary BSI domains and the GSI scale of the “Drug dependence” and the “No drug dependence” groups. The differences between the two groups were all highly significant (*p* < .001): drug-dependent prison inmates scored higher than non-drug-dependent inmates in every domain and, consequently, manifested a higher overall psychological distress level. The largest differences were measured in the hostility (*r* = 0.56) and depression (*r* = 0.58) domains and in the GSI (*r* = 0.51) scale. The remaining domains all had medium effect sizes (*r* ≥ 0.3), proving a medium to strong significant difference between the two groups.

**Table 2.** Comparison between the “drug dependence” and “no drug dependence” groups as represented by their means (*M*) in each subscale, standard deviation (*SD*), number of respondents in each group (*n*), the statistical significance (*p*), *z*-values, and effect size (*r*).

BSI domains	Drug dependence group	No drug dependence group	*p*	*z*	*r*
	*M*/*SD* (*n*)	*M*/*SD* (*n*)			
Anxiety	1.50/0.84 (58)	0.92/0.69 (66)	<.001	5.28	0.47
Somatization	1.34/0.72 (59)	0.81/0.72 (63)	<.001	4.14	0.37
Psychoticism	1.63/0.85 (55)	1.00/0.71 (62)	<.001	4.63	0.43
Paranoid ideation	1.88/0.89 (59)	1.12/0.83 (65)	<.001	5.03	0.45
Obsessive-compulsiveness	1.94/0.73 (56)	1.30/0.72 (66)	<.001	4.73	0.43
Hostility	1.50/0.83 (58)	0.70/0.59 (67)	<.001	6.28	0.56
Phobic anxiety	0.96/0.67 (58)	0.54/0.56 (66)	<.001	4.62	0.41
Depression	1.67/0.85 (57)	0.77/0.69 (68)	<.001	6.44	0.58
Interpersonal sensitivity	1.69/1.09 (62)	0.93/0.84 (68)	<.001	4.69	0.41
GSI	1.56/0.66 (36)	0.92/0.58 (50)	<.001	4.77	0.51

## Discussion

The present study is one of the first—to our knowledge—describing prison inmates’ psychological distress in Morocco. The study provides further contribution to previous studies (Alves et al., 2013; Gunter et al., 2008; Otte et al., 2017) on prison populations’ psychological distress. The distress level of the GSI alongside the nine primary symptom dimensions in Moroccan inmates was first presented, and then compared the level of psychological distress between “Drug dependence” and “No drug dependence” groups of inmates.

### General Psychological Distress Level in Moroccan Inmates

Moroccan inmates’ psychological distress level, measured by the General Severity Index (GSI), was comparable with the information of a nonprobability sample of 263 prisoners in a medium-security state prison in eastern Kentucky (Edwards & Potter, 2004) (American study GSI = 1.13), while was higher than it was found in medium- and high-security Swedish prisons’ population (Sfendla et al., 2018a) (Swedish study GSI = 1). We do not have data from the general Moroccan population of adults to compare with our findings.

Concerning BSI domains, the paranoid ideation scores among Moroccan prison inmates were the highest among other domain’s scores. When comparing the score of paranoid ideation to data from other countries’-such as Sweden (Sfendla et al., 2018a), Portugal (Alves et al., 2013), and Germany (Otte et al., 2017) -prison inmates, the Moroccan inmates notably reported the highest score.

The surveyed Moroccan prison inmates also reported a higher prevalence of psychotic and obsessive symptoms than Swedish prisoners (Sfendla et al., 2018a) and male Portuguese prisoners (Alves et al., 2013). These differences may be explained by the fact that some countries (like Sweden and Portugal) perform forensic psychiatric assessments of not yet convicted offenders (Dressing & Salize, 2006; Svennerlind et al., 2010). In such countries, offenders diagnosed with a severe mental disorder tend to be sentenced to forensic psychiatric care instead of prison; therefore, it could be assumed that there are fewer inmates with severe psychiatric symptoms in these countries’ prisons. On the contrary, in developing countries prisons often replace the role of mental hospitals and forensic psychiatric care. This may explain why psychotic symptoms were more frequently reported among Moroccan prison inmates in our study when compared to the prevalence of psychotic symptoms reported of prison inmates in Sweden (Sfendla et al., 2018a).

The higher level of psychological distress in Moroccan prison inmates may also be explained by the differences between prison conditions in developing and developed countries. Prisons that can offer their prisoners meaningful activities and/or treatment programs, such as those in developed countries, tend to have a prison population with lower psychological distress levels (Khabbache et al., 2017; Kjelsberg et al., 2006; McIntosh & Saville, 2006). However, prisons in developing countries are poorly equipped to provide inmates with health care services, which may lead to further impairments in their mental health (Fatoye et al., 2006). Morocco, for example, generally has a large shortage of psychiatrists, clinical psychologists, and nurses specializing in psychiatry (Khabbache et al., 2017). The presence of specialized medical health workers in prisons is sketchy (Gharaibeh & El-Khoury, 2009). Furthermore, in 2006, the Moroccan prison population grew twice as big as its infrastructure capacity could withhold. Aggravated conditions by overcrowded prison environments result in malnutrition, poor hygiene, and higher psychological distress (Gharaibeh & El-Khoury, 2009). Our results, therefore, are in line with previous findings suggesting that mental complaints increase in deteriorate prison conditions (Gharaibeh & El-Khoury, 2009; World Health Organization, 2005).

### Level of Psychological Distress in Prisoners with or without Drug Dependence

Moroccan prison inmates who, according to the Arabic DUDIT, can be classified as drug dependent, reported significantly higher levels of psychological distress compared to the inmates who were not classified as drug dependent. As the DUDIT assesses drug use habits over the past 12 months, and as we could not control how long the respondents had been incarcerated, the explanation of these significant differences between drug-dependent and non-drug-dependent prisoners can be approached from different angles.

It can be discussed how abstinence can affect the psychological distress level. It is possible that some of the members of the “Drug dependence” group were suffering from withdrawal due to not being able to pursue their substance use (at all or as regularly as before) inside the prison. Some of the signs of experiencing opioid withdrawal, for instance, include being introvert, depressed, and reporting low self-esteem (American Addiction Centers, 2018). Although there is consensus as regards the fact that increased anxiety, hostility, impulsivity, and depressive symptoms are common in inmates with a history of drug abuse (Værøy, 2011; Værøy et al., 2016), some studies have also demonstrated that abstinence is also usually coupled to trait anxiety, hostility, impulsivity and lacking a sense of coherence and that this association is not straight forward and is related to the duration of abstinence (Chen, 2009; Dennis et al., 2007; McCormick & Smith, 1995). The longer the duration of abstinence from substance use, the greater the improvement of the person’s mental health status tends to be (Dennis et al., 2007).

However, it is also essential to consider the possibility that drug-dependent inmates may actively abuse illicit drugs in the Moroccan prison environment. Opioids, such as heroin, tramadol, morphine, and codeine, are reported to be common among Moroccan prison inmates, but the prevalence of drugs in this prison environment remains unclear (National Observatory on Drugs and Addiction, 2014).

The results indicate that the “Drug dependence” group of prisoners reported a significantly higher level of GSI when compared to the “No drug dependence” group. While it is well known that substance abuse and/or dependency has a robust negative effect on a person’s mental health, it is also known that people with mental disorders are more prone to abuse substances (Schanda et al., 2004). The present study did not measure clinical diagnoses of mental disorders; instead, these diagnoses were operationalized into psychological distress level. High self-perceived psychological distress maybe both the reason for and the consequence of substance use.

Prisoners endure much pressure caused by the prison environment. Incarceration itself has a negative impact on prisoners’ mental health, and they are likely to feel depressed (Birmingham, 2003). Nonetheless, it was recently shown that those prisoners and outpatients who have depression have 17-times greater odds of being drug dependent (Sfendla et al., 2018b). One inevitable thing is that substance abuse is a contributing factor to mental ill-health, regardless of whether or not depression symptoms or substance use were the primary issues.

Studies have shown that depression is a risk factor for committing violent acts, and that substance use increases the risk of hostile behavior (Fazel et al., 2015; Iversen et al., 2014; Schanda et al., 2004). The fact that hostility and depression are often comorbid may explain why these two domains were distinguished as being increased with the largest effect size in “Drug dependent” inmates. Although the BSI’s hostility domain does not measure committed violent acts *per se*, it does measure violent thoughts and urges, which indicates that violence and hostility are related.

Substance use and failure to control hostile responses to provocation have been found to occur together (McCormick & Smith, 1995). Substance use may therefore be a contributing factor to why inmates express increased hostility. Research suggests that inmates with aggressive/hostile behavior often find themselves in difficult situations (coupled to negative emotions, social, and even physical conflicts), which can trigger drug abuse (Friedmann et al., 2008). For instance, it has been established that individuals with high levels of hostility find it more difficult to abstain from drugs at times of stress (Handelsman et al., 2000; McCormick & Smith, 1995). The findings of the present study among Moroccan inmates cannot confirm the causality effect of substance use on negative reactions, aggressive behaviors, impulsivity and irritability. However, they show a significantly increased psychological distress level, including hostility, in the “Drug dependence” group compared to the “No drug dependence” group of prison inmates. As DUDIT measures drug use habits during the past 12 months, it is the other possibility that the “Drug dependence” group of prisoners had been in the abstinence phase for less than 12 months, and therefore still had higher levels of irritability and hostility than the prisoners who had abstained for over 12 months or who had never been drug users. A previous study found that male inmates who had abstained for more than 12 months demonstrated lower levels of hostility when compared to those who had abstained for less than 12 months (Chen, 2009).

### Limitations

This study has methodological limitations that should be taken into account when interpreting its findings. A cross-sectional design was followed in this study. One limitation of cross-sectional studies resides in the fact that no follow-up about the inmate’s longitudinal development over time is possible; therefore, it is impossible to infer any causation effects (Billhult, 2017). The study was based on self-reported assessments, meaning that the results are dependent on the participants’ memory and honesty when answering the questionnaires (Secades-Villa & Fernández-Hermida, 2003; Voluse et al., 2012). It is also important to remember that the BSI is not used as a diagnostic tool and there is lack of BSI general population scores and norms.

Consequently, any comparison between our results and previous research, which mostly report the prevalence of clinically diagnosed disorders, should be made with caution. Finally, we had access only to male prison facilities, where the participation was voluntary among a suggested sample selected by the prison’s administration. Therefore, the results’ generalizability is low and may suggest that psychological distress is even higher in Moroccan prison inmate populations. We were unable to define the type of convictions and the length of sentences (to be able to keep the anonymity of the survey), both of which could affect the results since it was previously shown that psychological distress is linked both to the type of conviction and to the stage of sentences (Otte et al., 2017).

## Conclusion

Moroccan male prison inmates’ psychological distress level was comparable or higher than it was found in other countries’ prison population. When comparing the “Drug dependence” and “No drug dependence” groups, the result showed that drug dependence is associated with significantly higher psychological distress levels, especially in the hostility and depression domains. Further studies should focus on describing mental health in Moroccan general and prison populations. Only then can research examine the contribution of prison to the high psychological distress level of Moroccan prisoners and focus on changes to follow policy recommendation (Enggist et al., 2014) in accordance with the Sustainability Development Goals (UN, 2020).
